# A genome-wide association study reveals novel genomic regions and positional candidate genes for fat deposition in broiler chickens

**DOI:** 10.1186/s12864-018-4779-6

**Published:** 2018-05-21

**Authors:** Gabriel Costa Monteiro Moreira, Clarissa Boschiero, Aline Silva Mello Cesar, James M. Reecy, Thaís Fernanda Godoy, Priscila Anchieta Trevisoli, Maurício E. Cantão, Mônica Corrêa Ledur, Adriana Mércia Guaratini Ibelli, Jane de Oliveira Peixoto, Ana Silvia Alves Meira Tavares Moura, Dorian Garrick, Luiz Lehmann Coutinho

**Affiliations:** 1 Department of Animal Science, University of São Paulo (USP) / Luiz de Queiroz College of Agriculture (ESALQ), Piracicaba, São Paulo 13418-900 Brazil; 2 Department of Animal Science, Iowa State University (ISU), Ames, Iowa USA; 3 Embrapa Suínos e Aves, Concórdia, Santa Catarina Brazil; 4 FMVZ / São Paulo State University, Botucatu, SP Brazil; 5 School of Agriculture, Massey University, Ruakura, Hamilton New Zealand

**Keywords:** Genomic heritability, QTL, Abdominal fat, Skin weight, Fatness

## Abstract

**Background:**

Excess fat content in chickens has a negative impact on poultry production. The discovery of QTL associated with fat deposition in the carcass allows the identification of positional candidate genes (PCGs) that might regulate fat deposition and be useful for selection against excess fat content in chicken’s carcass. This study aimed to estimate genomic heritability coefficients and to identify QTLs and PCGs for abdominal fat (ABF) and skin (SKIN) traits in a broiler chicken population, originated from the White Plymouth Rock and White Cornish breeds.

**Results:**

ABF and SKIN are moderately heritable traits in our broiler population with estimates ranging from 0.23 to 0.33. Using a high density SNP panel (355,027 informative SNPs), we detected nine unique QTLs that were associated with these fat traits. Among these, four QTL were novel, while five have been previously reported in the literature. Thirteen PCGs were identified that might regulate fat deposition in these QTL regions: *JDP2*, *PLCG1*, *HNF4A*, *FITM2*, *ADIPOR1*, *PTPN11*, *MVK*, *APOA1*, *APOA4, APOA5,* ENSGALG00000000477, ENSGALG00000000483, and ENSGALG00000005043*.* We used sequence information from founder animals to detect 4843 SNPs in the 13 PCGs. Among those, two were classified as potentially deleterious and two as high impact SNPs.

**Conclusions:**

This study generated novel results that can contribute to a better understanding of fat deposition in chickens. The use of high density array of SNPs increases genome coverage and improves QTL resolution than would have been achieved with low density. The identified PCGs were involved in many biological processes that regulate lipid storage. The SNPs identified in the PCGs, especially those predicted as potentially deleterious and high impact, may affect fat deposition. Validation should be undertaken before using these SNPs for selection against carcass fat accumulation and to improve feed efficiency in broiler chicken production.

**Electronic supplementary material:**

The online version of this article (10.1186/s12864-018-4779-6) contains supplementary material, which is available to authorized users.

## Background

The chicken was the first domesticated animal species that was whole-genome sequenced and it has emerged as an excellent model for genomic studies in agriculture, developmental biology, fatness and leanness [1]. The main fat deposits in chicken are located in the skin (including subcutaneous fat) and within the abdominal cavity (abdominal plate) [2–4]. Excess fat deposition in broiler chickens is a negative factor for the poultry industry because it decreases feed efficiency and reduces the nutritional value of carcass parts and, consequently, their commercial value [5–7].

Broiler chicken lines have been selected for rapid growth, and carcass yield [7, 8]. Rapid growth results in increased fat deposition within the carcass [8] and commercial chickens exhibit higher fat deposition compared with unselected chickens [7]. The selection of chickens for rapid growth and reduced carcass fat deposition is challenging because these two traits have a positive genetic correlation [8].

Some studies have been conducted to map genomic quantitative trait loci (QTLs) associated with variation in abdominal fat [5, 9–13], and skin traits [9, 14]. However, most previously published QTLs were mapped using low density of markers (ranging from 102 to 410 markers), and the detected intervals spanned tens of centimorgans (cM) [15].

Previous genome-wide association studies (GWAS) have been performed for abdominal fat weight and fat percentage in an F2 chicken population’s using a 60 K SNP chip (Illumina) [16, 17] and in a local population of a local Chinese breed using approximately 90,000 SNPs [18]. To the best of our knowledge, no GWAS was reported for fatness traits in a meat-type population using the high-density SNP chip (600 K) from Affymetrix [19].

Fat deposition is an economically-relevant trait in fast-growing chickens, and knowledge about the genetic regulation of this trait is essential for breeding programs. Based on this fact, the main goal of this study was to perform GWAS analysis using a high-density SNP panel (600 K) to identify QTLs and positional candidate genes (PCGs) and possibly candidate mutations for fat deposition in broiler chickens.

## Methods

All experimental protocols related to animal experimentation in this study were performed in agreement with resolution number 011/2010 approved by the Embrapa Swine and Poultry Ethics Committee on Animal Utilization (CEUA) in Concordia, Santa Catarina State – South of Brazil, in agreement with the rules of National Council of Animal Experimentation Control (CONCEA) to ensure compliance with international guidelines for animal welfare.

### Chicken population

This study was conducted with a paternal broiler line (TT) belonging to the Chicken Breeding Program of EMBRAPA Swine and Poultry National Research Center, in Concordia, Santa Catarina State – South of Brazil. This line, originating from the White Plymouth Rock and White Cornish breeds, has been under multiple trait selection since 1992 mainly for body weight, feed conversion, carcass and cuts yield, viability, fertility, hatchability and reduced abdominal fat [13, 20–23]. The TT Reference Population evaluated in this study was developed in 2008 and consisted of 1430 chickens (652 males and 778 females) generated in five hatches from 20 males and 92 females (1:5). Previous genomic studies have been performed in this population, and more details can be found in [20–23].

### Phenotype measurement

After 6 h of fasting, the chickens at 42 days of age were weighted (BW42) and then euthanized by cervical dislocation. In this step, a blood sample from each chicken was immediately collected for subsequent DNA extraction then, the carcass was cooled. After 4 h of cooling, the weights of the carcass, skin covering each carcass part (thigh, drumstick, and breast) and abdominal fat (abdominal fat pad) were measured. The percentage of each trait was calculated dividing the weigh by BW42 and multiplying by 100. Total skin weight and percentage were used as indicators of subcutaneous fat, as discussed by Zerehdaran et al. [3]. More details about the rearing conditions and phenotypes measurements are available in Fornari et al. [22].

### DNA extraction, genotyping and quality control

Genomic DNA from 1430 blood samples were extracted using the PureLink® Genomic DNA (Invitrogen, Carlsbad, CA, USA) kit and were quantified using Qubit® 2.0 Fluorometer (Thermo Fisher Scientific, Waltham, MA, USA). After extraction, DNA integrity was evaluated on agarose gel (1%) and diluted to 10 ng/μL. Diluted genomic DNA was prepared following recommended Affymetrix protocols in order to perform the genotyping analysis using 600 K Affymetrix Axiom Genotyping Array (Affymetrix, Inc. Santa Clara, CA, USA). This genotyping array comprises segregating SNPs for different chicken populations, including four commercial broilers (broiler chicken lines), as detailed by Kranis et al. [19].

Initially, Axiom™ Analysis Suite (Affymetrix®) software was used to filter genotypes based on the DishQC parameter, after which PLINK v.1.9 [24] software was used to perform quality control analysis and for genotype calling. Only samples that exhibited DishQC ≥0.82 and call rate ≥ 90% were kept for further analysis. Considering these retained samples, in order to select markers with high quality, and to avoid potential genotyping errors or even DNA contamination, further edits were undertaken based on literature recommendations [25], to remove single nucleotide polymorphisms with a call rate ≤ 98%, minor allele frequency (MAF) ≤ 2% or significant deviations from HWE (*p*-value < 0.000001). Single nucleotide polymorphisms located in the sex chromosomes, and those SNPs not mapped in the chicken assembly (Gallus_gallus-5.0, NCBI) were excluded from the analysis. Only the SNPs annotated to autosomal chromosomes from GGA1 to GGA28 were used in statistical analyses. After all the filtering steps, the few remained missing genotypes were replaced by the average of covariate values at that particular locus, as described by Cesar et al. [26].

From a total of 1430 genotyped chickens, 22 samples were removed from the analysis after applying the DishQC criteria, and a filter on sample call rate ≥ 90% loci. From the total of 580,961 SNPs available on the SNP array, 355,027 informative polymorphic SNPs on the autosomal chromosomes (GGA1–28) were kept after filtering. The average density of SNPs was 520 SNPs/Mbp, with the lowest chromosome-wise density observed on GGA2 (268 SNPs/Mbp), and the highest chromosome-wise density on GGA21 (898 SNPs/Mbp) (Additional file 1).

### Descriptive statistics and heritability

The mean and the standard deviation of each phenotype were calculated using R scripts. The estimation of variance components (genetic variance, residual variance, and total variance) was performed using a Bayes C model in GenSel software [27] using the samples and SNPs that remained after genotyping and filtering. The resultant posterior means of the variance components were used as *priors* in subsequent Bayes B models to estimate genomic heritability for each trait.

### Genome wide association analysis

The SNPs that passed the quality control filters were used in the GWAS analysis using genomic prediction methodology with a Bayesian approach in GenSel software [27]. In the first step, a Bayes C model was used to estimate the genetic and residual variances for each trait and these values were then used as *priors* to run a Bayes B model as performed by Cesar et al. [26]. The Bayes B models sample the effects of SNPs assuming some fraction of the effects are zero and with unequal variance of each effect [28]. The mathematical model was:

$$ \boldsymbol{y}=\boldsymbol{Xb}+\sum \limits_{j=1}^k{\boldsymbol{a}}_j{\beta}_j{\delta}_j+\boldsymbol{e}, $$

In this model, ***y*** represents the vector of phenotypic values; ***X*** is the incidence matrix for fixed effects; ***b*** is the vector of fixed effects; *K* is the number of SNPs; a_*j*_ is the column vector representing the SNP as a covariate in locus _j_ coded with the number of B alleles; *βj* is the random substitution effect for locus *j* assumed to be normally distributed *N* (0, *σ*^2^_*βi*_) when *δj* = 1 but *βj* = 0 when *δj* = 0, with *δj* being a random variable 0/1 indicating the absence (with probability π) or presence (with probability 1-π) of the locus *j* in the model, and ***e*** is the residual associated with the analysis. Sex and hatch were included as fixed effects in the model and BW42 (slaughter age) as a fixed covariate for ABF and SKINW.

We assumed π = 0.9970 in the BayesB models and obtained 41,000 Markov chain Monte Carlo (MCMC) samples with the first 1000 samples being discarded. A map file was used to position the markers into 947 non-overlapping 1 Mb windows. The windows that had the marker with higher model frequency in the MCMC interactions had their effect predicted as mentioned by Van Goor et al. [29]. Each window is expected to explain 0.1054% of the genetic variance (100%/947) based on an infinitesimal model [30, 31], and windows that explained five times more than the expected (0.53%) were considered significant. Thus, we selected only significant windows to characterize and identify PCGs.

### Overlap with known QTLs

The overlap of our genomic windows with previously mapped QTLs for fat-related traits in chickens was determined using the information available at Chicken QTLdb - release 33, accessed in September, 2017 [32]. We used the available BED file with the QTL coordinates according to the last chicken genome assembly (Gallus_gallus-5.0, NCBI) to check the overlaps using in-house R scripts. The genomic windows that did not overlap with previously annotated QTLs for fat traits were considered to be novel discoveries. All the previously mapped QTLs were reported by QTL ID numbers, available at Chicken QTLdb [32].

### Identification of positional candidate genes

A list of annotated genes within each QTL (genomic window) and their respective GO terms and biological processes were obtained using Ensembl BioMart tool [33, 34]. Genes that had GO terms and a biological process related to fat deposition were initially selected. Next, two different databases (NCBI, OMIM) were searched to identify existing literature to support/refute the positional candidate genes (PCG) identified.

Enrichment analysis of gene list was performed with the Functional Annotation Tool (FAT) in Database for Annotation, Visualization and Integrated Discovery software (DAVID bioinformatics resources v.6.8, [35, 36]) to identify enriched clusters of genes. To select a gene cluster as enriched, we considered an enrichment score > 1.00 and within the cluster, GO terms for biological process (BP) with a raw *p*-value < 0.05 and p-value adjusted for multiple testing by Benjamini & Hochberg [37] method < 0.1.

### Candidate genes screening for SNPs from sequencing data

To refine our list of candidate genes, we screened our PCGs for genetic variants using a dataset of SNPs identified in the parental generation from our genotyped population, which were generated by next generation sequencing of 14 parental males (from 112) with approximately 13 X of coverage performed by HiScanSQ (Illumina) with a read length of 101 bp. Further details about library preparation and sequencing are available in Moreira et al. [38] and Godoy et al. [39].

SNP calling was performed using the most recent chicken genome assembly Gallus-gallus-5.0 (UCSC) with SAMtools software v.1.2 [40], with mapping and base qualities (Phred score) ≥ 20. The filtering criteria and further details about SNP calling are available in Boschiero et al. [41]. After variant filtering, the SNP dataset was annotated using Variant Effect Predictor (VEP) tool version 86 [42]. SNP density were determined considering all the unique SNPs annotated (including 5 Kb up and downstream) in each PCG and its gene length.

Variants located in coding regions can lead to phenotypic changes [38, 39, 43]. To predict whether SNPs that caused changes in amino acids were tolerant or not (may affect the function of the gene product), we calculated the SIFT (sorting intolerant from tolerant) score. This score is an assessment of the level of conservation in homologous protein sequences using the SIFT algorithm [44] implemented by the VEP tool version 86 [42]. SIFT scores were calculated for all the non-synonymous and stop codon (gained/lost) variants located in the PCGs.

High impact SNPs were also evaluated in the candidate genes. The VEP tool [42] provides an estimation of the putative impact of the variant classified as high impact, i.e. annotating all the mutations annotated as transcript ablation, splice acceptor, splice donor, stop gained, frameshift, stop loss, start lost and transcript amplification, mutations that may cause protein truncation, loss of function or trigger nonsense mediated decay [43].

## Results

### Descriptive statistics and genomic heritability

The number of animals, averages and standard errors, variance components and estimated genomic heritability from the Bayes B model are given in Table 1 for ABF, ABFP, SKINW and SKINP. We estimated genomic heritability values to be moderate for all traits evaluated; ABF traits exhibited higher genomic heritability compared to SKIN traits.

**Table 1:** Descriptive statistics, variance components and genomic heritability for body weight at 42 days of age, abdominal fat and skin weights and percentages in the TT Reference Population

Trait	N	Average ± SD^a^	Genetic variance	Residual Variance	Total variance	Genomic heritability^b^
BW42	1311	2220.30 ± 258.86	12,378.000	25,423.100	37,801.100	0.33
ABF	1287	47.10 ± 14.03	46.599	96.079	142.677	0.33
ABFP	1287	2.13 ± 0.62	0.094	0.205	0.299	0.31
SKINW	1303	94.55 ± 16.12	29.936	96.443	126.379	0.23
SKINP	1303	4.25 ± 0.56	0.063	0.203	0.266	0.23

BW42: body weight at 42 days of age in grams; *ABF* abdominal fat weight in grams, *ABFP* abdominal fat percentage, *SKINW* skin weight in grams, *SKINP* skin percentage

^a^Standard deviation of the mean

^b^Genomic heritability estimated by Bayes B model

### Genome wide association analysis (GWAS)

A list with all the SNP windows analyzed including the proportion of the genetic variance explained by each one (even those with effects close to zero) is provided in Additional file 2. The QTLs (significant genomic windows) associated with fat deposition are described in Table 2. Nine unique significant 1 Mb windows (with different unique positions) were identified on GGA 5, 9, 10, 13, 15, 20, 24, 26, and 27. The posterior probability of association (PPA), as described by Onteru et al. [31], ranged from 0.82 to 0.95 for each region, and the proportion of genetic variance explained by the window ranged from 0.54 to 1.49.

**Table 2:** Characterization of 1 Mb significant genomic windows for abdominal fat and skin traits in the TT Reference Population

Trait	GGA (Mb)^a^	SNP window (first – last position)^a^	Number of SNP/ window	Number of genes/ window^b^	Proportion of genetic variance explained by the SNP window	PPA^c^
ABF	5 (38)	38,000,437–38,996,916	396	31	0.92	0.84
	10 (7)	7,000,336–7,998,549	592	21	0.58	0.93
	13 (3)	3,002,617–3,998,616	460	16	1.45	0.88
	20 (5)	5,000,651–5,999,452	492	53	0.94	0.88
	26 (1)	1,002,598–1,999,851	662	74	1.06	0.95
ABFP	5 (38)	38,000,437–38,996,916	396	31	0.64	0.82
	10 (7)	7,000,336–7,998,549	592	21	0.61	0.90
	13 (3)	3,002,617–3,998,616	460	16	1.49	0.89
	26 (1)	1,002,598–1,999,851	662	74	0.54	0.92
SKINW	15 (6)	6,000,311–6,999,944	544	62	0.73	0.89
	24 (5)	5,000,105–5,999,010	778	60	0.56	0.91
	27 (3)	3,000,222–3,997,124	933	52	0.60	0.94
SKINP	9 (4)	4,000,836–4,999,336	482	50	0.73	0.83
	15 (6)	6,000,311–6,999,944	544	62	0.71	0.91
	27 (3)	3,000,222–3,997,124	933	52	0.57	0.95

*ABF* bdominal fat weight, *ABFP* abdominal fat percentage, *SKINW* skin weight, *SKINP* skin percentage

^a^Map position based on Gallus_gallus-5.0 assembly (NCBI)

^b^Number of genes annotated within the genomic window based on *Ensembl Genes 90 Database*

^c^Posterior probability of association (PPA) as described by Onteru et al. [31]

**Fig. 1:**
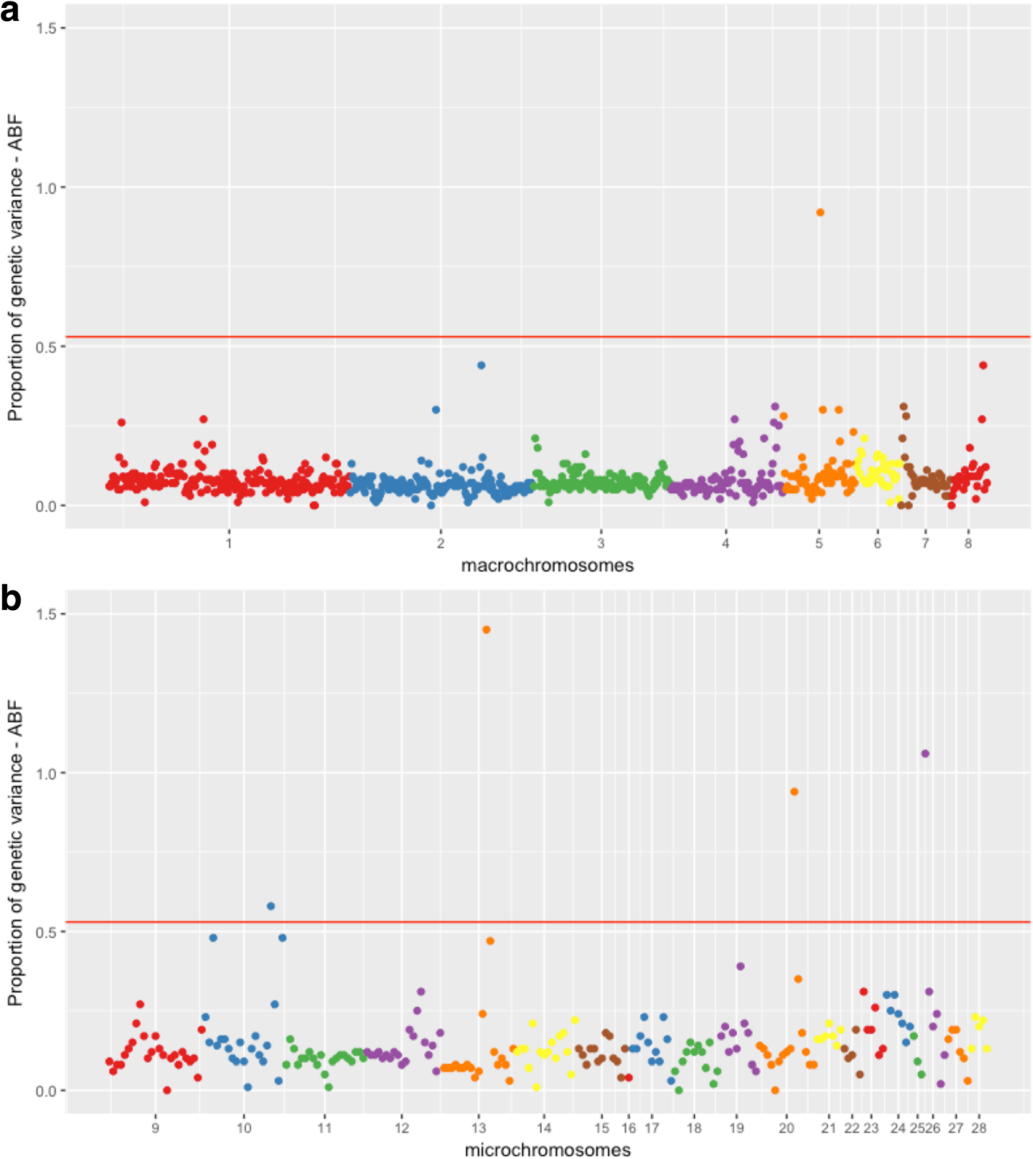
Manhattan plot of the posterior means of the proportion of genetic variance explained by each 1-Mb SNP window across the 28 autosomal chromosomes for abdominal fat weight (ABF): (**a**) genomic windows located on macrochromosomes, and (**b**) windows located on microchromosomes. The X-axis represents the chromosomes, and Y-axis shows the proportion of genetic variance explained by each window from Bayes B analysis. Red lines indicate the threshold to deem significant SNP windows

The Manhattan plot of the posterior means of the proportion of genetic variance explained by each SNP window across the 28 autosomal chromosomes for ABF are presented in Fig. 1. The Manhattan plots for ABFP, SKINW and SKINP are in Additional files 3, 4, and 5, respectively.

**Fig. 2:**
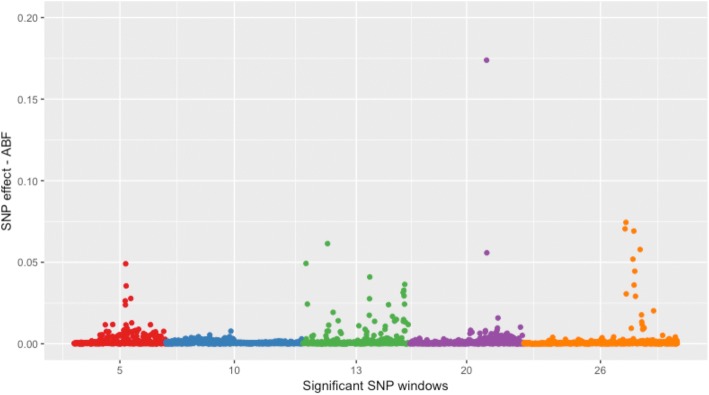
Manhattan plot of the SNP effect distribution within each significant window for abdominal fat weight (ABF). The X-axis represents the significant SNP window represented by the number of the respective chromosome and Y-axis shows the SNP effect from Bayes B analysis. Their respective start and end positions are: GGA5 (38,000,437–38,996,916 bp); GGA10 (7,000,336–7,998,549 bp); GGA13 (3,002,617–3,998,616 bp); GGA20 (5,000,651–5,999,452 bp); GGA26 (1,002,598–1,999,851 bp)

In order to support our findings, we checked the effect of the markers within the associated genomic windows. Manhattan plots of the SNP effect distribution within each significant SNP window for ABF are colored by chromosome and presented in Fig. 2. The Manhattan plots for ABFP, SKINW and SKINP are in Additional files 6, 7, and 8, respectively.

### Overlap with previously reported QTLs

Twenty-seven previously published QTLs for fat traits overlapped with five of the QTLs identified in our study. The QTL located on GGA5 at 38 Mb, associated with ABF and ABFP overlapped with three known QTLs: two QTLs associated with for ABF (QTL #3321, [45]; QTL #9432, [46]) and one associated with ABFP (QTL #9433, [46]).

The QTL, which was located on GGA15 at 6 Mb and associated with SKINW and SKINP, overlapped with 11 QTLs previously associated with fat traits: four were associated with for ABF (QTL #2337, [5]; QTL #9451, [46]; QTL #2347, QTL #12631, [9]), three were associated with ABFP (QTL #2339, QTL #2340, [5]; QTL #9450, [46]), one associated with fat distribution (total weight of skin fat analyzed with ABF as covariate) (QTL #12645, [9]), one QTL associated with subcutaneous neck fat weight (QTL #17331, [2]), one QTL associated with total white fat weight (QTL #17337, [2]), and one associated with visceral fat weight (QTL #17323, [2]).

The QTL located on GGA24 at 5 Mb that was associated with SKINW, overlapped with one QTL previously reported to be associated with ABF (QTL #9405, [47]). While, the QTL identified on GGA26 at 1 Mb that was associated with ABF and ABFP, overlapped with two QTLs: one associated with visceral fat weight (QTL #17324, [2]), and one associated with total white fat weight (QTL #17338, [2]).

The QTL identified on GGA27 at 3 Mb that was associated with SKINW and SKINP, overlapped with 10 QTLs previously reported to be associated with fat traits: three associated with ABF (QTL #66072, [48]; QTL #11817, QTL #11809, [49]), three associated with ABFP (QTL #11820, [49]; QTL #3354, [50]; QTL #11934, [51]), two associated with carcass fat content (QTL #17135, QTL #17126, [13]), one associated with carcass fat content on a dry matter basis (QTL #17125, [13]), and one associated with intramuscular fat (QTL #3360, [50]).

No previously reported QTL overlapped with the QTL identified on GGA9 at 4 Mb, GGA10 at 7 Mb, GGA13 at 3 Mb, and GGA20 at 5 Mb.

### Positional candidate genes

We identified 419 genes in the nine QTL genomic windows (Additional file 9). Further analysis against gene ontology terms and the existing literature revealed 13 candidate genes for fat deposition (Table 3).

**Table 3:** List of candidate genes within the genomic windows associated with abdominal fat and skin traits that exhibited GO terms related to lipid metabolic processes in the TT Reference Population

GGA (location, Mb)	Trait associated	Gene Name	Ensembl Gene ID	GO Term (GO Accession)^a^
5 (38)	ABF, ABFP	*JDP2*	ENSGALG00000010322	negative regulation of fat cell differentiation (GO:0045599)
15 (6)	SKINW, SKINP	*PTPN11*	ENSGALG00000004821	lipid metabolic process (GO:0006629)
				triglyceride metabolic process (GO:0006641)
		*MVK*	ENSGALG00000013848	lipid metabolic process (GO:0006629)
				cholesterol metabolic process (GO:0008203)
		Novel gene	ENSGALG00000005043	fatty acid biosynthetic process (GO:0006633)
				acetyl-CoA carboxylase activity (GO:0003989)
20 (5)	ABF	*FITM2*	ENSGALG00000026285	lipid storage (GO:0019915)
				lipid particle organization (GO:0034389)
		*PLCG1*	ENSGALG00000003750	lipid metabolic process (GO:0006629)
				lipid catabolic process (GO:0016042)
		*HNF4A*	ENSGALG00000004285	lipid metabolic process (GO:0006629)
				regulation of lipid metabolic process (GO:0019216)
				lipid homeostasis (GO:0055088)
				fatty acid binding (GO:0005504)
24 (5)	SKINW	*APOA4*	ENSGALG00000007109	lipid homeostasis (GO:0055088)
				multicellular organismal lipid catabolic process (GO:0044240)
				positive regulation of triglyceride catabolic process (GO:0010898)
				cholesterol homeostasis (GO:0042632)
				cholesterol metabolic process (GO:0008203)
				positive regulation of fatty acid biosynthetic process (GO:0045723)
		*APOA5*	ENSGALG00000014368	triglyceride homeostasis (GO:0070328)
				positive regulation of lipid catabolic process (GO:0050996)
				positive regulation of fatty acid biosynthetic process (GO:0045723)
		*APOA1*	ENSGALG00000007114	lipid transport (GO:0006869)
				lipid metabolic process (GO:0006629)
				lipid storage (GO:0019915)
				cholesterol homeostasis (GO:0042632)
26 (1)	ABF, ABFP	Novel gene	ENSGALG00000000477	lipid metabolic process (GO:0006629)
				lipid catabolic process (GO:0016042)
				lipid particle (GO:0005811)
				lipid homeostasis (GO:0055088)
		Novel gene	ENSGALG00000000483	lipid catabolic process (GO:0016042)
				lipid particle (GO:0005811)
				lipid homeostasis (GO:0055088)
				triglyceride lipase activity (GO:0004806)
				triglyceride catabolic process (GO:0019433)
		*ADIPOR1*	ENSGALG00000000094	regulation of lipid metabolic process (GO:0019216)
				fatty acid oxidation (GO:0019395)

^a^All GO terms were obtained from BioMart (*Ensembl Genes 90 Database*)

Additionally, all 419 genes located within the detected QTLs were used to perform enrichment analysis. One cluster was enriched (enrichment score of 2.62) and within this cluster, four GO terms were enriched: regulation of intestinal cholesterol absorption; high-density lipoprotein particle assembly, lipoprotein metabolic process and positive regulation of fatty acid biosynthetic process (raw *p*-value < 0.05 and p-value adjusted for multiple testing by Benjamini & Hochberg [37] method < 0.1). These GO terms were annotated for the same genes: *APOA1*, *APOA4*, and *APOA5.*

### SNPs in candidate genes

A previous study has been performed using sequencing data to identify and characterize genome-wide SNPs, INDELs, putative regions under selection, and also to find putative pathways under selection in two Brazilian chicken lines [41], but neither was based on the TT broiler reference population.

**Fig. 3:**
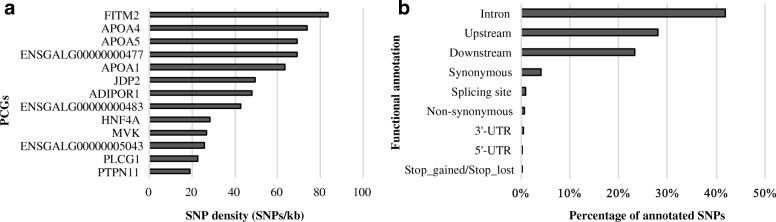
**a** Plot of SNP density (SNPs/kb) for each PCG. **b** Plot with the percentage of functional annotation of SNPs identified in our 13 PCGs

We used a dataset of high quality SNPs from sequencing data identified in 14 parental chickens from TT Reference Population in order to screen for SNPs potentially affecting gene expression and/or function and identified 3639 SNPs located within the 13 PCG. SNP density (SNPs/kb) within each PCG and the functional annotation of the SNPs are presented in Fig. 3. The PCG that had the greatest density of SNPs was *FITM2* (84 SNPs/kb).

Single nucleotide polymorphisms were evaluated for potentially deleterious and high impact mutation annotation, which may potentially affect gene expression and/or function. Two high impact variants were identified: one located within *FITM2* and another located in ENSGALG00000000483 gene. In addition, two potentially deleterious variants were identified: one located in *PLCG1* and another in ENSGALG00000000477 gene (Table 4). The non-synonymous SNP located in the *PLCG1* gene is novel.

**Table 4:** Characterization of potentially deleterious and high impact SNPs identified in the 13 PCGs

Associated Gene Name	Ensembl Gene ID	SNP ID	SNP Position^a^	Consequence
*FITM2*	ENSGALG00000026285	rs315805239	5,614,711	Stop gained
Novel gene	ENSGALG00000000483	rs740555722	1,514,268	Stop loss
*PLCG1*	ENSGALG00000003750	g.5072909A > T	5,072,909	Non-synonymous
Novel gene	ENSGALG00000000477	rs737351616	1,509,828	Non-synonymous

^a^Position based on Gallus_gallus*-*5.0 assembly

## Discussion

### Genomic heritability

Genomic heritability estimates for abdominal fat and skin traits in a broiler chicken population characterized by close relatives (full and half-sibs) were obtained using genotypes from a high-density SNP panel. Close relatives may have long chromosome segments in common, thereby sharing alleles and QTLs in the same pattern, which may lead to less bias and consequently, higher prediction accuracy for genomic heritability [52].

Heritabilities estimates for ABF and ABFP have been reported to be 0.62 and higher, while the heritability of skin traits is between 0.24 and 0.28 [3, 53]. The number of generations of artificial selection and/or the genetic background may differ for each chicken population thus, different heritability estimates may be observed. The TT broiler line, used to obtain the TT Reference population, has been under multi-trait selection with emphasis on body weight. This trait has a positive genetic correlation with abdominal fat and feed conversion [54]. Therefore, artificial selection may also affect the genetic variance and may reduce heritability over the generations [55]. Comparisons between heritabilities reported in the literature should be interpreted with caution.

Using the same population reported here, Fornari et al. [22] observed similar pedigree-based heritability estimates to those we obtained using genomic information, namely for abdominal fat weight (0.33) and two skin related traits: drumstick skin weight (0.17) and thigh skin weight (0.28). The existence of moderate genomic heritabilities for the analyzed phenotypes indicates that a reasonable proportion of the total variance for these traits can be explained by a set of markers [52]. Therefore, selection against fat deposition in this population may be successful.

### GWAS, QTL discovery and resolution

Bayesian approaches are commonly used in genomic prediction and selection studies [56, 57] as well as for GWAS [29, 30, 58, 59] in chickens. The main advantage of this approach is that genotypes are simultaneously fitted in the model, accounting for population structure, and the use of high-density markers does not reduce the power to detect association [60]. Thus, we decided to use genomic prediction methodology to perform GWAS.

Five out of the nine QTLs detected were previously identified in different populations, corroborating our results, and indicating that these QTLs probably originated from the founder lines used to generate the broiler TT line used in this study.

The novel QTLs identified could be false positives, exclusive to our population, or a consequence of the number of animals and the higher SNP density compared to other QTL mapping studies for abdominal fat and skin traits in chickens [5, 8–14]. The PPA (ranging from 0.83 to 0.93) and the proportion of the genetic variance explained by the novel QTLs (ranging from 0.58 to 1.49) suggest that these novel QTL are not false positives. Besides that, the Manhattan plots of the SNP effects within the QTLs detected showed few peaks indicating that some markers exhibit higher effects within the QTL (see the direction of the SNP effects; Fig. 2, Additional files 6, 7, and 8) providing helpful information for further studies aiming to fine-map these QTLs.

In general, we observed small effects for the markers fitted simultaneously within the QTLs detected. This could be due to lack of power to capture the genetic variability in our population or, due to artificial selection. As mentioned before, the artificial selection can lead to fixation of specific loci [55, 61] and SNPs with higher effect on fatness may have been fixed over the generations.

In contrast to the QTL mapped on GGA5 at 38 Mb, previously reported QTLs for the same trait were larger than 1 Mb [45, 46]. The use of a higher density of markers (600 K) may help to explain the improved resolution observed in QTL mapping.

The QTL mapped on GGA27 at 3 Mb overlapped with known QTLs for fatness related traits, mapped in a Brazilian F2 population established by crossing a broiler male line (TT) and a layer line (CC), and these known QTLs are segregating in different families from the Brazilian F2 population [13, 49]. Furthermore, the broiler male line (TT) used in the crossing to establish this population, is the same line used to obtain the TT Reference Population [13] thus, we should expect this QTL segregating in our broiler population, corroborating our findings.

We identified only a few QTLs associated with fat traits in this population. For quantitative traits, a greater number of alleles are expected to present a small effect [62], and the number of samples used in this study may not have been sufficiently large enough to identify these small effect QTLs. Despite this, novel QTLs for fat traits in broiler were identified. These QTLs should be considered as novel QTLs may be population-specific.

### Positional candidate genes for fat deposition

We identified 13 PCG in five of the nine QTLs identified (Table 3). In the QTL on GGA5 at 38 Mb we identified the *Jun dimerization protein 2* (*JDP2*) gene. This gene regulates lipid accumulation in adipose tissue acting as a repressor of adipocyte differentiation [63, 64].

We identified *PLCG1*, *HNF4A* and *FITM2* genes in the QTL on GGA20 at 5-Mb. In human primary adipocytes, *Phospholipase C gamma 1* (*PLCG1*) gene is involved in the calcium signaling pathway. The expression of *PLCG1* has been show to affect adipocyte triglyceride content [65]. Hepatocyte nuclear factor-4α (*HNF4A*) controls insulin metabolism and triglycerides level [66]. Triglycerides are the main lipid stored in avian fat cells. Thus, different levels of plasma triglyceride may affect fat deposition [67]. The *Fat storage inducing transmembrane protein 2* (*FITM2/FIT2*) gene belongs to a family of proteins that play a role in fat storage [68]. In a study with humans, the *FITM2/FIT2* gene was reported to be associated with lipid droplets biogenesis and accumulation [69], which consequently, impacts lipid storage.

In the QTL on GGA26 at 1 Mb, we found *ADIPOR1*, ENSGALG00000000477 and ENSGALG00000000483 genes. In chickens, the adiponectin receptor 1 (*ADIPOR1*) gene is expressed in fat, liver and muscle, and this gene affect adipocyte differentiation [70]. *ADIPOR1* is the main receptor of adiponectin. It is negatively correlated with fat deposition [70], and is involved in lipid-induced mitochondrial biogenesis in chicken adipocytes [71]. ENSGALG00000000477 and ENSGALG00000000483 code for uncharacterized proteins, but their gene ontologies are related to lipid metabolism and storage (Table 3). Further studies with those novel genes may help elucidate their role in fat deposition.

In the QTL on GGA15 at 6 Mb, we found *PTPN11, MVK* and ENSGALG00000005043 genes. The *protein tyrosine phosphatase, non-receptor 11* (*PTPN11*) gene encodes for a Src homology-2 domain-containing protein tyrosine phosphatase 2 (*SHP2*). Its expression has been reported to affect energy balance and lipid and glucose metabolisms [72]. Additionally, in a study with mice, *SHP2* was reported to be associated with obesity [73]. Mevalonate kinase (*MVK*) encodes for a mevalonate kinase enzyme that plays an important role at the beginning of cholesterol biosynthesis [74]. Changes in the cholesterol biosynthesis, and consequently cholesterol levels, may affect hepatic lipid metabolism [75]. ENSGALG00000005043 is a novel gene that has been annotated with GO term related to the fatty acid biosynthetic process and Acetyl-CoA carboxylase activity (Table 3). Further studies with these genes may help to better understand their role in fat deposition.

In the QTL located on GGA24 at 5 Mb we found *APOA1*, *APOA4* and *APOA5* genes. These three genes belong to a gene family (Apolipoproteins – APO) that encodes important regulators of lipid biosynthesis and metabolism [76]. Additionally, these three positional genes were annotated with four enriched GO terms: regulation of intestinal cholesterol absorption, high-density lipoprotein particle assembly, lipoprotein metabolic process, and positive regulation of the fatty acid biosynthetic process. Apolipoprotein A1 (*APOA1*) is involved with cholesterol transport [77]. While, *Apolipoprotein A-IV* (*APOA4*) and *Apolipoprotein V* (*APOA5*) are involved with triglycerides metabolism [76]. Additionally, the *APOA4* gene was also reported as a regulator of triglycerides metabolism in mice [77].

Corroborating our findings, no overlap between our positional candidate genes, and genes under selective pressure reported in a previous study with the same dataset [23] was observed.

Additionally, comparing a dataset of SNPs and INDELs identified in Brazilian broiler and layer lines, our group recently identified regions under selection [41], harbouring genes related to fat deposition. No overlap was observed between our PCGs for fat deposition and the genes reported in that study, except for *APOA1*. Possible explanations for the observed lack of overlap, are the different chicken lines used in these studies, and the removal of fixed SNPs in the current study.

### Potential causative SNPs in PCGs

We observed many SNPs annotated in intronic regions of the PCGs (approximately 42% of the SNPs; Fig. 3b). According to the literature, introns can play a role in the regulation of alternative splicing, gene expression, and may be associated with mRNA transport [78, 79]. Thus, SNPs annotated in introns can play a role in the regulation of any trait, including fat deposition in chickens.

Approximately 58% of the SNPs found in the 13 PCG were in potentially functional regions such as up/downstream, 3’and 5’-UTRs, exons (synonymous and non-synonymous), splicing site and stop codon (gained/lost; Fig. 3b). Genetic variants within non-coding regions (3’and 5’-UTRs) may control gene expression by modulating transcription or mRNA turnover [80]. We observed 36 SNPs in 3/5’-UTR regions (Fig. 3b). Two of the 35 non-synonymous SNPs were classified as potentially deleterious and were located in *PLCG1* and ENSGALG00000000477 genes (Table 4), PCGs for fat deposition regulation. Potentially deleterious SNPs in these genes could be causative mutations.

Two high impact SNPs were annotated in *FITM2* and ENSGALG00000000483 genes (Table 4). High impact SNPs in these genes may affect lipid metabolism and storage (fat deposition) in chickens.

From the four SNPs (Table 4), one is novel (g.5072909A > T), and the others are not included on the Affymetrix SNP array. Thus, the integration of GWAS and genome sequencing brought additional information in the search for potential causative mutations. Further studies are necessary to achieve a better understanding of the role of these SNPs in fat deposition.

## Conclusions

This study confirmed previously published QTLs and discovered novel ones, thus contributing to a better understanding of fat deposition in chickens. The use of a high-density SNPs panel in our GWAS analyses provided a better resolution in QTL detection. The PCGs identified in the QTL are involved in many biological processes that regulate lipid storage. We found SNPs located in the PCGs providing additional information in the search for potential causative mutations and further validation studies could be helpful to understand their role in fat deposition regulation. Our findings can be potentially applied to improve the accuracy of early selection against carcass fat accumulation and improve feed efficiency in broiler chicken production.

## Additional files

Additional file 1: Plot of the density of SNPs per Mbp in each autosomal chromosome after filtration. (DOCX 155 kb)

Additional file 2: An excel file with the characterization of all the SNP windows analyzed including the proportion of the genetic variance explained by each one. (ODS 137 kb)

Additional file 3: Manhattan plot of the posterior means of the proportion of genetic variance explained by each 1-Mb SNP window across the 28 autosomal chromosomes for abdominal fat percentage (ABFP): (A) genomic windows located on macrochromosomes, and (B) windows located on microchromosomes. The X-axis represents the chromosomes and Y-axis shows the proportion of genetic variance explained by each window from Bayes B analysis. Red lines indicate the threshold to deem significant SNP windows. (DOCX 245 kb)

Additional file 4: Manhattan plot of the posterior means of the proportion of genetic variance explained by each 1-Mb SNP window across the 28 autosomal chromosomes for skin weight (SKINW): (A) genomic windows located on macrochromosomes, and (B) windows located on microchromosomes. The X-axis represents the chromosomes and Y-axis shows the proportion of genetic variance explained by each window from Bayes B analysis. Red lines indicate the threshold to deem significant SNP windows. (DOCX 243 kb)

Additional file 5: Manhattan plot of the posterior means of the proportion of genetic variance explained by each 1-Mb SNP window across the 28 autosomal chromosomes for skin percentage (SKINP): (A) genomic windows located on macrochromosomes, and (B) windows located on microchromosomes. The X-axis represents the chromosomes and Y-axis shows the proportion of genetic variance explained by each window from Bayes B analysis. Red lines indicate the threshold to deem significant SNP windows. (DOCX 243 kb)

Additional file 6: Manhattan plot of the SNP effect distribution within each significant window for abdominal fat percentage (ABFP). The X-axis represents the significant SNP window represented by the number of the respective chromosome and Y-axis shows the SNP effect from Bayes B analysis. Their respective start and end positions are: GGA5 (38,000,437–38,996,916 bp); GGA10 (7,000,336–7,998,549 bp); GGA13 (3,002,617–3,998,616 bp); GGA26 (1,002,598–1,999,851 bp). (DOCX 1425 kb)

Additional file 7: Manhattan plot of the SNP effect distribution within each significant window for skin weight (SKINW). The X-axis represents the significant SNP window represented by the number of the respective chromosome and Y-axis shows the SNP effect from Bayes B analysis. Their respective start and end positions are: GGA15 (6,000,311–6,999,944 bp); GGA24 (5,000,105–5,999,010 bp); GGA27 (3,000,222–3,997,124 bp). (DOCX 1425 kb)

Additional file 8: Manhattan plot of the SNP effect distribution within each significant window for skin percentage (SKINP). The X-axis represents the significant SNP window represented by the number of the respective chromosome and Y-axis shows the SNP effect from Bayes B analysis. Their respective start and end positions are: GGA9 (4,000,836–4,999,336 bp); GGA15 (6,000,311–6,999,944 bp); GGA27 (3,000,222–3,997,124 bp). (DOCX 1425 kb)

Additional file 9: List with the 419 genes annotated within the nine QTL detected. (XLSX 61 kb)

## Abbreviations

ABF: Abdominal Fat Weight
ABFP: Abdominal Fat Percentage
BW42: Body Weight at 42 days of age
DAVID: Database for Annotation, Visualization and Integrated Discovery software
GWAS: Genome-Wide Association Study
HWE: Hardy Weinberg Equilibrium
MAF: Minor Allele Frequency
MCMC: Markov Chain Monte Carlo
NCBI: National Center for Biotechnology Information
OMIM: Online Mendelian Inheritance in Man
PCG: Positional Candidate Gene
PPA: Posterior Probability of Association
QTL: Quantitative Trait Locus
SKINP: Skin Percentage
SKINW: Skin Weight
