# Supplementary material for: A genome-wide association study reveals novel genomic regions and positional candidate genes for fat deposition in broiler chickens
Source: BMC Genomics. 2018 May 21;19:374. doi: 10.1186/s12864-018-4779-6 (PMC5963092; doi:10.1186/s12864-018-4779-6)

Additional file 1 - Plot of the density of SNPs per Mbp in each autosomal chromosome after filtration.


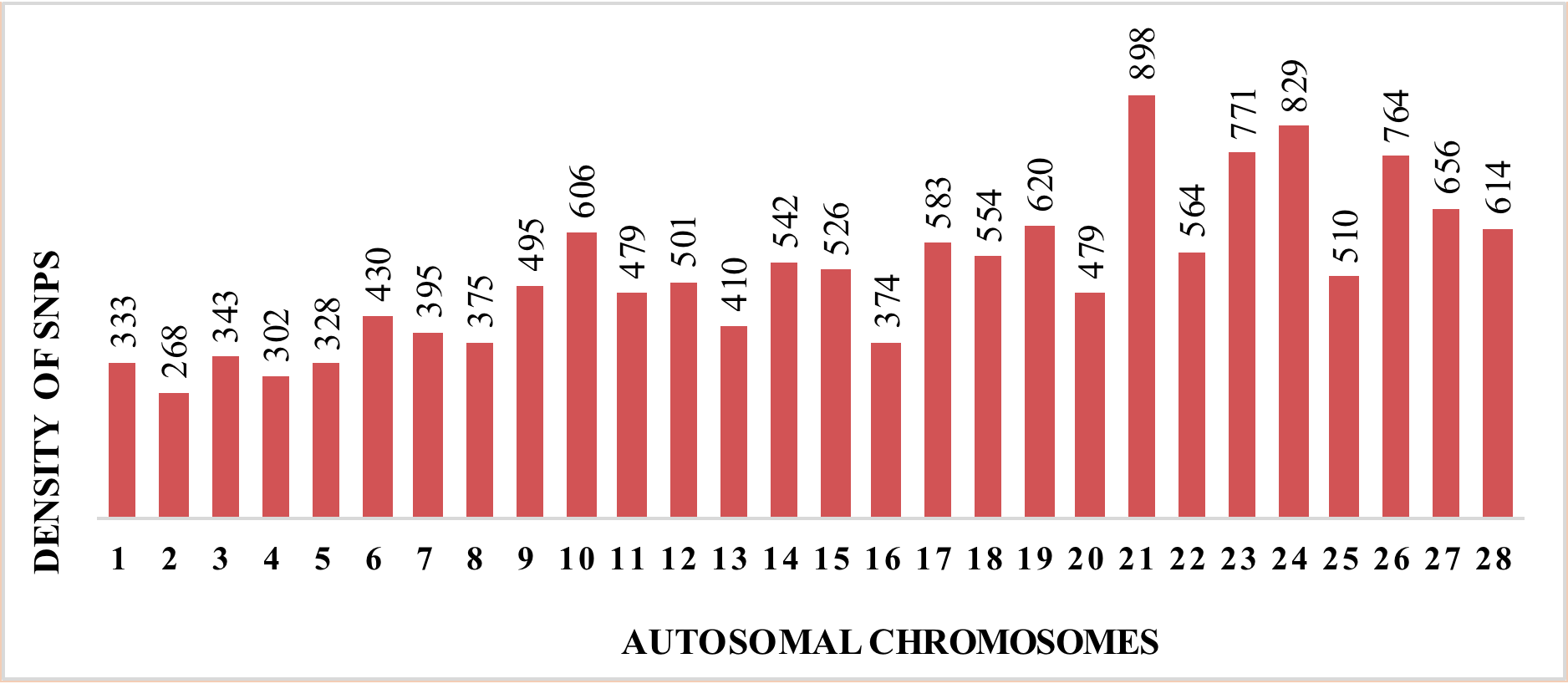

Supplement: Supplementary file 1 — Plot of the density of SNPs per Mbp in each autosomal chromosome after filtration. (DOCX 155 kb) [file 12864_2018_4779_MOESM1_ESM.docx]
