# Supplementary material for: A genome-wide association study reveals novel genomic regions and positional candidate genes for fat deposition in broiler chickens
Source: BMC Genomics. 2018 May 21;19:374. doi: 10.1186/s12864-018-4779-6 (PMC5963092; doi:10.1186/s12864-018-4779-6)
Supplement: Supplementary file 4 — Manhattan plot of the posterior means of the proportion of genetic variance explained by each 1-Mb SNP window across the 28 autosomal chromosomes for skin weight (SKINW): (A) genomic windows located on macrochromosomes, and (B) windows located on microchromosomes. The X-axis represents the chromosomes and Y-axis shows the proportion of genetic variance explained by each window from Bayes B analysis. Red lines indicate the threshold to deem significant SNP windows. (DOCX 243 kb) [file 12864_2018_4779_MOESM4_ESM.docx]

Additional file 4 – Manhattan plot of the posterior means of the proportion of genetic variance explained by each 1-Mb SNP window across the 28 autosomal chromosomes for skin weight (SKINW): (A) genomic windows located on macrochromosomes, and (B) windows located on microchromosomes. The X-axis represents the chromosomes and Y-axis shows the proportion of genetic variance explained by each window from Bayes B analysis. Red lines indicate the threshold to deem significant SNP windows.
