# Supplementary material for: A genome-wide association study reveals novel genomic regions and positional candidate genes for fat deposition in broiler chickens
Source: BMC Genomics. 2018 May 21;19:374. doi: 10.1186/s12864-018-4779-6 (PMC5963092; doi:10.1186/s12864-018-4779-6)

Additional file 6 – Manhattan plot of the SNP effect distribution within each significant window for abdominal fat percentage (ABFP). The X-axis represents the significant SNP window represented by the number of the respective chromosome and Y-axis shows the SNP effect from Bayes B analysis. Their respective start and end positions are: GGA5 (38,000,437 – 38,996,916 bp); GGA10 (7,000,336 – 7,998,549 bp); GGA13 (3,002,617 – 3,998,616 bp); GGA26 (1,002,598 – 1,999,851 bp).


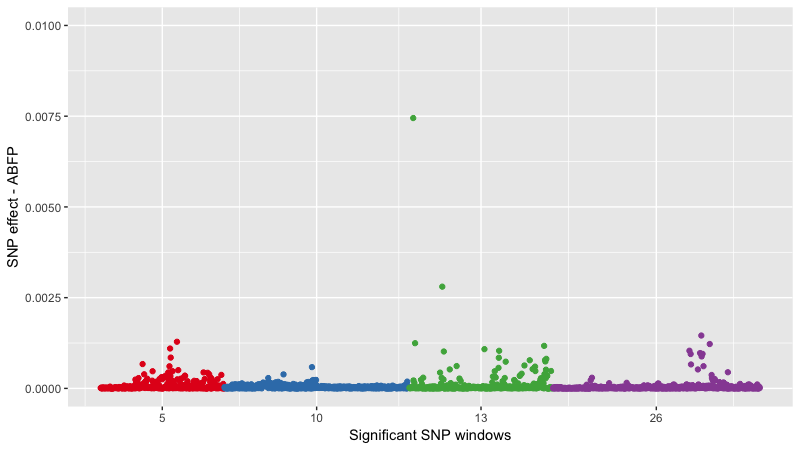

Supplement: Supplementary file 6 — Manhattan plot of the SNP effect distribution within each significant window for abdominal fat percentage (ABFP). The X-axis represents the significant SNP window represented by the number of the respective chromosome and Y-axis shows the SNP effect from Bayes B analysis. Their respective start and end positions are: GGA5 (38,000,437–38,996,916 bp); GGA10 (7,000,336–7,998,549 bp); GGA13 (3,002,617–3,998,616 bp); GGA26 (1,002,598–1,999,851 bp). (DOCX 1425 kb) [file 12864_2018_4779_MOESM6_ESM.docx]
