# Supplementary material for: A genome-wide association study reveals novel genomic regions and positional candidate genes for fat deposition in broiler chickens
Source: BMC Genomics. 2018 May 21;19:374. doi: 10.1186/s12864-018-4779-6 (PMC5963092; doi:10.1186/s12864-018-4779-6)

Additional file 8 – Manhattan plot of the SNP effect distribution within each significant window for skin percentage (SKINP). The X-axis represents the significant SNP window represented by the number of the respective chromosome and Y-axis shows the SNP effect from Bayes B analysis. Their respective start and end positions are: GGA9 (4,000,836 – 4,999,336 bp); GGA15 (6,000,311 – 6,999,944 bp); GGA27 (3,000,222 – 3,997,124 bp).


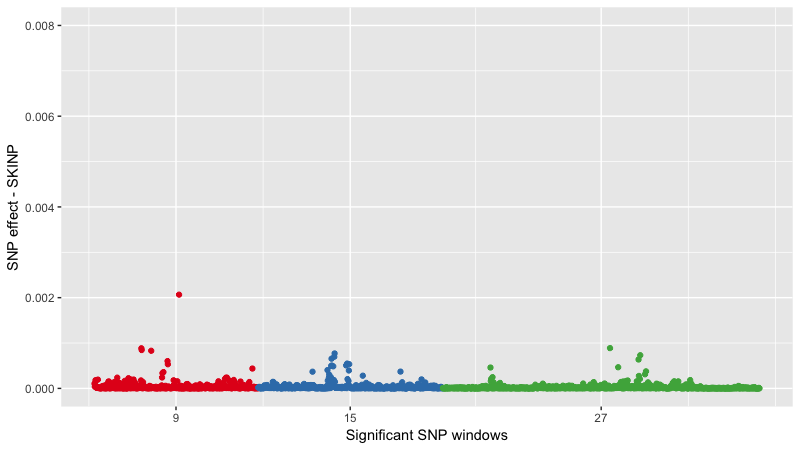

Supplement: Supplementary file 8 — Manhattan plot of the SNP effect distribution within each significant window for skin percentage (SKINP). The X-axis represents the significant SNP window represented by the number of the respective chromosome and Y-axis shows the SNP effect from Bayes B analysis. Their respective start and end positions are: GGA9 (4,000,836–4,999,336 bp); GGA15 (6,000,311–6,999,944 bp); GGA27 (3,000,222–3,997,124 bp). (DOCX 1425 kb) [file 12864_2018_4779_MOESM8_ESM.docx]
